# Sexual dysfunction in Spanish women with breast cancer

**DOI:** 10.1371/journal.pone.0203151

**Published:** 2018-08-31

**Authors:** Ana Isabel Cobo-Cuenca, Noelia María Martín-Espinosa, Antonio Sampietro-Crespo, María Aurora Rodríguez-Borrego, Juan Manuel Carmona-Torres

**Affiliations:** 1 Departamento de Enfermería, Fisioterapia y Terapia Ocupacional, E.U. Enfermería y Fisioterapia de Toledo, Universidad de Castilla la Mancha (UCLM), Toledo, Spain; 2 Instituto Maimónides de Investigación Biomédica de Córdoba (IMIBIC), Córdoba, Spain; 3 Hospital Virgen de la Salud, Toledo, Spain; 4 Departamento de Enfermería, Facultad de Medicina y Enfermería, Universidad de Córdoba, Córdoba, Spain; 5 Hospital Universitario Reina Sofía de Córdoba, Córdoba, Spain; Universidad Miguel Hernandez de Elche, SPAIN

## Abstract

**Purpose:**

To determine whether there are changes in sexuality after breast cancer, to better understand the sexual function of women with breast cancer, and to investigate the potential relationship between sexual dysfunction and socio-demographic and clinical variables.

**Methods:**

A cross-sectional study. This study included 514 women with breast cancer between 21- and 66-years-old. The cases were gathered between June 2016 and January 2017. The instruments used were the questionnaire on Women’s Sexual Function and a questionnaire to collect socio-demographic and clinical data.

**Results:**

The average age (± standard deviation, SD) of participants was 46.34 ± 8.28 years. Their average age at date of diagnosis was 42.26 ± 8.56 years, and the average time suffering from cancer was 4.05 ± 5.23 years. There were significant differences (p = 0.002) in the presence of sexual dysfunction before (32.1%) and after (91.2%) cancer. The primary sexual dysfunctions were due to penetration pain (50.6%), lubrication (50.6%), dysfunctional desire (44.6%), and dysfunctional excitement (44.6%). Two-thirds of participants were satisfied with their sexual relations. The women who presented most sexual dysfunction were those that had a bilateral mastectomy (p = 0.009) and those who received chemotherapy, radiotherapy and hormonal-therapy (p < 0.001).

**Conclusion:**

Sexual function was changed in women with breast cancer. The main problems included penetration pain, desire, lubrication, and dysfunctional excitement. It is important that Health professionals recognize which circumstances influence the sexual function of women with breast cancer and to make interventions that facilitate sexual adjustment.

## Introduction

Breast cancer is the second most common cancer worldwide. In 2012, the estimated number of new cases was 1.67 million (25% of all cancers). In Western Europe, there are 96 per 100,000 women with breast cancer [1].

According to the Spanish Society for Medical Oncology [2], the cancer incidence in Spain during the year 2015 was 247,771 cases (148,827 men and 98,944 women). Breast cancer has a greater incidence (27,747), a greater prevalence over 5 years, and a greater mortality rate (6,213 deaths during 2014) than other cancers [3]. The prevalence of breast cancer is increasing due to increased life expectancy and advanced diagnostic techniques for cancer [2, 3, 4].

The Spanish healthcare system is public with universal access. This system includes programs for the prevention, screening, and treatment of breast cancer [4]. Furthermore, there are associations that offer support to patients with cancer. For breast cancer, the most important association is the Spanish Federation of Breast Cancer (FECMA, by its initials in Spanish). FECMA has 27 centers in Spain.

Breast cancer treatment affects women’s sexual function. These treatments, which include hormonal therapy, chemotherapy, and surgical treatment, can produce vaginal dryness, vasomotor symptoms, and loss of sexual satisfaction, loss of desire, and changes in self-image [5,6,7]. Studies addressing women’s suffering from breast cancer have found that between 30 and 70% of patients present sexual dysfunction [5, 6, 7].

In Spain, no recent studies have addressed sexual dysfunction in women with breast cancer. Sexual function depends on several biological, psychological, and sociocultural factors. Here we investigate sexuality in women with breast cancer in Spain.

The aims of this study were: to determine if there are changes in the sexuality of women after breast cancer; to understand the sexual function of women with breast cancer in Spain; and to describe the relationship between socio-demographic and clinical variables with sexual dysfunction.

## Methods

### Participants and design

This is a cross-sectional study carried out from June 2016 to January 2017. The population of the study consisted of 514 women with breast cancer who belonged to FECMA. Inclusion criteria: women with breast cancer, 18-years-old or older, and completed chemotherapy, radiotherapy or surgical intervention at least 3 months before the present study. Exclusion criteria: women with other cancers, metastases, and terminal conditions.

### Sample size

The sample size was determined using Granmo software (version 7.12, Barcelona, Spain, 2012). This was calculated using a population estimate based on the study carried out by Panjari et al. (2011) [6], in which 70% of women with breast cancer suffered from sexual dysfunction, as well as the data offered by the Spanish Society for Medical Oncology [2] in which 27,747 women had breast cancer in Spain. Therefore, a sample of 355 women would be expected to be sufficient for an expected prevalence of 70%, a precision of 5%, a level of confidence of 95%, and a replacement rate of 10%.

### Instruments

An online questionnaire was used to collect the different socio-demographic and clinical variables. This online questionnaire also included the *Questionnaire on Women's Sexual Function (WSF)* of Sánchez et al. (2004) [8]. This scale was created and validated to evaluate sexual function in Spanish women. It is a self-administered scale consisting of 14 items with a Likert scale with five options. Women were asked to answer in relation to the past 4 weeks. The first six questions refer to the different phases of the sexual response. Questions 7 and 8 assess the relational aspects of sexual activity. Questions 9 and 10 assess sexual satisfaction. The remaining questions address relevant aspects of sexual activity. The cut-points for the score range were: desire, excitation or arousal, penetration pain [severe problem (1–3), moderate problem (4–7), without problem (8–15)]; sexual satisfaction [no satisfaction (1–2), moderate satisfaction (3–5), satisfaction (6–10)]; lubrication, orgasm, and anticipatory anxiety [severe problem (1), moderate problem (2), without problem (3–5)]; sexual initiative [abscense of initiative (1), moderate initiative (2), without problem (3–5)]; and communication [absence of sexual communication (1), moderate sexual communication (2), without problem (3–5)]. This scale demonstrated an internal consistency of 0.895–0.897 and a reliability of 0.597–0.743 [8].

To determine whether the women had any sexual problem before cancer, the online survey had two questions: 1) “Before suffering cancer, have you had any sexual problem?”; 2) If your answer is “YES”, could you specify what type of sexual problem you have had?”

### Variable Sociodemographic and clinical

Age (quantitative), level of education (categorical), profession (categorical), civil status (categorical), type of surgical intervention (categorical), breast reconstruction (categorical), adjuvant treatment (categorical), sexual dysfunction before breast cancer (categorical), age of women at date of diagnosis (quantitative), and time elapsed after diagnosis (quantitative).

### Variable of type of sexual dysfunction

Desire, excitation or arousal, lubrication, orgasm, penetration pain, anticipatory anxiety, sexual initiative, communication and sexual satisfaction (categorical variables).

### Procedure

Before the study, the FECMA directors were contacted and invited to participate in the study. They sent to their members an email with information about the study. The email included a direct access link to fulfill the online questionnaire available on Google Forms. When clicking on the link, the participants had to read the information sheet and provide informed consent to proceed with the questionnaire. The application respected the participant’s anonymity.

This study conformed to the main principles of the Helsinki Declaration, the Universal Declaration of the UNESCO, and the Spanish Organic Law 15/1999, 13^th^ of December, on the Protection of Personal Data. The study was approved by FECMA. The participants were provided with an information sheet and gave informed consent. We respected the participants’ anonymity at all times. The Ethical Committee authorized this study (CEITO N° Exp 138).

### Data analysis

The statistics program IBM SPSS (version 22.0, licensed by the University of Castile La-Mancha, IBM Corp, Armonk, NY, USA) was used for statistical analysis. A descriptive analysis of the variables was performed by counting of recounts (n) and proportions (%) of the qualitative variables and by the average (m) and the standard deviation (SD) of quantitative variables.

Also, a proportional comparison of categorical variables through chi-square tests for contingency tables was provided. The variables, which were significates with sexual dysfunction in the contingency tables, were included in the regression model. A multiple logistic regression was performed to identify possible factors related to sexual dysfunction after suffering from breast cancer. We used the Wald statistic, such that the variables for which p ≥ 0.15 were eliminated one-by-one from the model. The odds ratios (OR) was calculated with their confidence intervals. All hypotheses were bilateral, and the level of confidence was 95% (p < 0.05).

## Results

Five-hundred-twenty-seven women responded to the questionnaire, 13 of these completed the test incorrectly or incompletely. Finally, 514 women with breast cancer composed the size. The study participants were aged between 21- and 66-years-old (mean 46.34 ± 8.276 years), an average age at the date of diagnosis of 42.26 ± 8.563 years, and time suffering cancer of 4.05 ± 5.226 years. Table 1 shows the socio-demographic characteristics related to cancer treatment.

**Table 1:** Socio-demographic and clinical variables of participants (n = 514).

Qualitative variables	Frequency (N)	Percentage (%)
Marital Status		
Single without partner	33	6.4
Single with long-term partner	47	9.2
Married women or living with a partner	346	67.3
Divorced women with long-term partner	28	5.4
Divorced women without partner	53	10.3
Widowed without long-term partner	4	0.8
Widowed with partner	3	0.6
Education		
Incomplete elementary	3	0.6
Elementary	61	11.8
High school	225	43.8
University	225	43.8
Occupation		
Unemployed	106	20.6
Employed	257	50
Sick leave	94	18.3
Retired	57	11.1
Surgical intervention		
Lumpectomy	195	37.9
Mastectomy	214	41.7
Bilateral Mastectomy	86	16.7
Lymphadenectomy	19	3.7
Adjuvant treatment		
Radiotherapy	12	2.3
Chemotherapy	30	5.8
Hormonal therapy	25	4.9
Radiotherapy and chemotherapy	63	12.2
Radiotherapy and HT	45	8.8
Chemotherapy and HT	64	12.5
Radiotherapy, chemotherapy and HT	254	49.4
No adjuvant treatment	21	4.1
Breast reconstruction		
Yes	208	40.5
No	185	36
Not necessary	121	23.5
Quantitative Variables	Mean (M)	Standard Deviation (SD)
Age	46.3	8.3
Age at diagnosis	42.3	8.6
Time elapsed following diagnosis	4.0	5.2

Regarding sexual activity (Table 2), the majority of women did not present any sexual dysfunction before breast cancer diagnosis (66.9%). However, after having breast cancer (when the data were collected), 91.2% suffered from some type of sexual dysfunction. There are significant differences (p = 0.002) between the presence of sexual dysfunction before and after cancer.

**Table 2:** Types of Sexual function of participants (n = 514).

Sexual Function	Frequency (N)	Percentage (%)
Sexual Dysfunction before cancer		
No	344	66.9
Yes	170	33.1
Sexual Dysfunction after cancer		
No	45	8.8
Yes	469	91.2
Dysfunction Desire after cancer		
Severe problem	43	8.4
Moderate problem	186	36.2
Without problem	285	55.4
Dysfunction Excitation after cancer		
Severe problem	57	11.1
Moderate problem	177	34.4
Without problem	280	54.5
Dysfunction due to Lubrication after cancer		
Severe problem	122	23.7
Moderate problem	138	26.9
Without problem	254	49.4
Orgasm Dysfunction after cancer		
Severe problem	121	23.5
Moderate problem	57	11.1
Without problem	336	65.4
Dysfunction due to penetration pain after cancer		
Severe problem	50	9.7
Moderate problem	210	40.9
Without problem	254	49.4
Dysfunction anticipatory Anxiety after cancer		
Severe problem	256	49.8
Moderate problem	86	16.7
Without problem	172	33.5
Initiative sexual after cancer		
Absence of initiative	232	45.1
Moderate Initiative	96	18.7
Without problem of initiative	186	36.2
Communication after cancer		
Absence of sexual communication	132	25.7
Moderate sexual Communication	80	15.5
Without problem of Sexual communication	302	58.8
Level of satisfaction of sexual activity		
No satisfaction	86	16.7
Satisfaction moderate	100	19.5
Satisfactory	328	63.8
Level of Sexual General Satisfaction		
Sexual general no satisfaction	94	18.3
Moderate Satisfaction	79	15.4
Satisfaction general	341	66.3
Motive no penetration		
Pain	63	30.3
Afraid to penetration	6	2.9
Lack of interest in vaginal penetration	71	34.1
Without sexual partner	54	26
Incapacity of sexual partner	14	6.7
Sexual activity		
Yes	361	70.2
No	153	29.8
Frequency		
1–2 times a month	312	60.7
3–4 times a month	110	21.4
5–8 times a month	69	13.4
9–12 times a month	23	4.5

Table 2 shows the types of sexual dysfunctions that women suffer after breast cancer. The most frequent dysfunctions were dysfunction due to penetration pain (50.6% of participants), dysfunction due to lubrication (50.6%), dysfunctional desire (44.6%), and dysfunctional excitation or arousal (44.6%). Regarding their sexual activity in the last 4 weeks, the majority usually had 1–2 instances of sexual activity per month (60.7%) and were satisfied with their sexual relations (66.3%). Finally, the most frequent reasons why these women did not want penetration during their sexual relations were a lack of interest in vaginal penetration (34.1%) and pain (30.3%).

The relationship between socio-demographic and clinical variables and the different types of sexual dysfunction in participants after cancer were studied (Table 3). We found that women who received adjuvant treatments, radiotherapy, and hormonal therapy had a higher percentage of sexual dysfunction (p < 0.001) compared to women who received other adjuvant treatments. Women who had a bilateral mastectomy (p = 0.009) had greater dysfunction than those who had a lumpectomy.

**Table 3:** Socio- demographic variables of participants and relation with dysfunction sexual after cancer (n = 514).

	Sexual dysfunction after cancer		p
Qualitative Variables	NO (n = 45) n (%)	YES (n = 469) n (%)	
Civil Status			0.138
Single without partner	3 (6.7)	30 (6.4)	
Single with long-term partner	2 (4.4)	45 (9.6)	
Married women/ living with a partner	28 (62.2)	318 (67.8)	
Divorced women with partner	6 (13.4)	22 (4.7)	
Divorced without partner	5 (11.1)	48 (10.2)	
Widowed without partner	0	4 (0.9)	
Widowed with partner	1 (2.2)	2 (0.4)	
Studios Level			0.003
Incomplete elementary	0	3 (0.6)	
Elementary	13 (28.8)	48 (10.2)	
High School	16 (35.6)	209 (44.6)	
University	16 (35.6)	209 (44.6)	
Occupation			0.414
Unemployed	8 (17.8)	98 (20.9)	
Employed	23 (51.1)	234 (49.9)	
Sick leave	6 (13.3)	88 (18.8)	
Retired	8 (17.8)	49 (10.4)	
Surgical Intervention			0.009
Lumpectomy	26 (57.8)	169 (36)	
Mastectomy	17 (37.8)	197 (42)	
Bilateral Mastectomy	2 (4.4)	84 (17.9)	
Lymphadenectomy	0	19 (4.1)	
Adjuvant Treatment			<0.001
Radiotherapy	5 (11.1)	7 (1.5)	
Chemotherapy	1 (2.2)	29 (6.2)	
Hormonal treatment	2 (4.4)	23 (4.9)	
Radiotherapy and chemotherapy	3 (6.7)	60 (12.8)	
Radiotherapy and hormonal treat.	9 (20)	36 (7.7)	
Chemotherapy and hormonal treat.	5 (11.1)	59 (12.6)	
Radiotherapy, chemotherapy and hormonal treatment	17 (37.8)	237 (50.5)	
Nor adjuvant treatment	3 (6.7)	18 (3.8)	
Reconstruction			0.593
Yes	15 (33.3)	193 (41.2)	
No	18 (40)	167 (35.6)	
Not necessary	12 (26.7)	109 (23.2)	
Quantitative Variables	NO M (SD)	YES M (SD)	
Age	49 (±7.3)	46.0 (±8.3)	0.006
Age at diagnosis	45.93 (±7.8)	41.94 (±8.5)	0.003
Time elapsed following diagnosis	3.64 (±4.0)	4.09 (±5.3)	0.584

Using the significant variables, we created a multiple logistic regression model to understand whether the variables affected every type of sexual dysfunction (Tables 4 and 5). In general, sexual dysfunction after cancer is associated with the age of women at the date of diagnosis (OR = 0.959; p = 0.029; 95%CI = 0.923–0.006) and surgical intervention. The probability of sexual dysfunction in women who have had a bilateral mastectomy is 4.684-times greater than those that had a lumpectomy (OR = 0.042; 95%CI = 1.056–20.784).

**Table 4:** Logistic regression of sexual dysfunction after cancer (n = 514).

	OR (95% CI)	p
Age at diagnosis	0.959 (0.923–0.996)	0.029
Surgical Intervention		
Lumpectomy	Reference	
Mastectomy	1.629 (0.847–3.133)	0.143
Mastectomy bilateral	4.684 (1.056–20.784)	0.042
Lymphadenectomy	22371 (0.007–0)	0.998

*Note*. Introduced variables: age at diagnosis, surgical intervention.

OR = Odds ratio; CI = confidence interval.

**Table 5:** Logistical regression of type sexual dysfunction (n = 514).

	Desire		Excitation		Lubrication		Orgasm		Penetration		Anxiety	
	OR (95% CI)	p	OR (95% CI)	p	OR (95% CI)	p	OR (95% CI)	p	OR (95% CI)	p	OR (95% CI)	p
*Age at diagnosis*	1.04 (1.02–1.07)	<0.001	1.04 (1.02–1.07)	0.001	1.06 (1.04–1.09)	<0.001	1.05 (1.02–1.08)	<0.001	1.03 (1.01–1.06)	0.006		
*Time elapsed f*. *diagnosis*					1.04 (0.99–1.08)	0.057			1.06 (1.02–1.10)	0.007	1.06 (1.02–1.11)	0.004
*Surgical Intervention*												
Lumpectomy	Reference		Reference		Reference		Reference					
Mastectomy	1.59 (0.92–2.76)	0.1	1.25 (0.83–1.88)	0.29	1.08 (0.72–1.62)	0.724	1.62 (0.91–2.87)	0.105				
Bilateral Mastectomy	2.88 (1.39–5.95)	0.004	2.02 (1.14–3.57)	0.015	2.30 (1.29–4.11)	0.005	2.93 (1.36–6.32)	0.006				
Lymphadenectomy	2.02 (0.76–5.36)	0.159	2.37 (0.86–6.52)	0.094	2.26 (0.81–6.31)	0.12	1.25 (0.45–3.44)	0.671				
*Reconstruction*												
Yes	Reference						Reference					
No	1.84 (1.13–2.98)	0.013					2.57 (1.54–4.29)	<0.001				
Not necessary	1.31 (0.65–2.63)	0.454					1.83 (0.87–3.86)	0.113				
*Civil Status*												
Single/divorced/widowed without partner	0.78 (0.48–1.26)	0.308	0.99 (0.62–1.61)	0.98	0.78 (0.48–1.26)	0.313	1.79 (1.09–2.92)	0.021	2.55 (1.54–4.21)	<0.001	1.05 (0.65–1.69)	0.84
Single/divorced/widowed with partner	0.52 (0.29–0.9)	0.02	0.45 (0.25–0.80)	0.006	0.51 (0.30–0.88)	0.015	0.77 (0.42–1.40)	0.338	1.18 (0.70–1.97)	0.54	2.01 (1.19–3.39)	0.009
Married women/living with a partner	Reference		Reference		Reference		Reference		Reference		Reference	
*Occupation*												
Unemployed			0.53 (0.27–1.05)	0.07							1.39 (0.71–2.74)	0.241
Employed			0.48 (0.26–0.90)	0.021							2.10 (1.14–3.86)	0.017
Sick leave			0.78 (0.39–1.58)	0.495							1.22 (0.60–2.48)	0.576
Retired			Reference								Rererence	
*Hormonal Treatment*												
No					Reference				Reference		Reference	
Yes					1.63 (1.06–2.51)	0.27			0.58 (0.38–0.88)		0.62 (0.41–0.95)	0.028

*Note*. Introduced variables = age at diagnosis, Time elapsed f diagnosis: time elapsed following diagnosis, Surgical intervention, reconstruction, civil status, occupation and hormonal treatment:

OR = Odds ratio; CI = confidence interval.

After analyzing the data related to the type of sexual dysfunction (Table 5), we observed the following:

Sexual dysfunction related to desire is associated with the age of women at date of diagnosis, the type of surgical intervention (women who had a bilateral mastectomy had a 2.28-fold greater probability of dysfunction than those who had a lumpectomy), breast reconstruction (women who did not receive breast reconstruction had a 1.84-fold greater probability than those who did) and cohabitation (women with a partner but who are not living together had a 0.54-fold reduction in probability compared to women living with a partner).

Sexual dysfunction related to excitation is associated with the age of women at date of diagnosis, the type of surgical intervention (women who had a bilateral mastectomy had a 2.02-fold greater probability than those who had a lumpectomy), cohabitation (women with a partner but who are not living together have a 0.45-fold reduction in probability compared to women living with a partner) and profession (women who work have a 0.48-fold reduction in probability than those who are retired).

Sexual dysfunction related to lubrication is associated with the age of women at date of diagnosis, the time elapsed after cancer diagnosis, the type of surgical intervention (women who had a bilateral mastectomy had a 2.30-fold greater probability than those that had a lumpectomy), cohabitation (women with a partner but who are not living together have a 0.51-fold reduction in probability compared to women living with a partner), and hormonal therapy (women who had hormonal therapy have a 1.63-fold increased probability compared to those who have not been treated with hormones).

Sexual dysfunction related to orgasm is associated with the age of women at date of diagnosis, the type of surgical intervention (women who had a bilateral mastectomy have a 2.93-fold greater probability than those who had a lumpectomy), breast reconstruction (women who have not received a breast reconstruction have a 2.57-fold greater probability than those who had), and cohabitation (women with a partner but who are not living together have a 1.79-fold increased probability compared to women living with a partner).

Sexual dysfunction related to penetration is associated with the age of women at date of diagnosis, the time elapsed after cancer diagnosis, cohabitation (women with a partner but who are not living together have a 2.55-fold reduction in probability compared to women living with a partner), and hormonal therapy (women who have had hormonal therapy have a 0.58-fold increased probability compared to those who have not been treated with hormones).

Anticipatory sexual anxiety is associated with the time elapsed after cancer diagnosis, cohabitation (women with partner but who are not living together have a 2.01-fold reduction in probability compared to women living with a partner), occupation (women who work have a 2.10-fold increase in probability than those who have retired), and hormonal therapy (women who have had hormonal therapy have a 0.62-fold reduction in probability compared to those who have not been treated with hormones).

## Discussion

According to other studies [6, 9, 10, 11, 12], women who have suffered from breast cancer report that their sexuality is changed. However, before cancer, 66.9% did not have any type of sexual difficulty. After breast cancer, 91.2% reported that they had some type of sexual difficulty. This incidence is higher than other studies, where it was found to vary from 40 to 80% [5, 6, 9, 13, 14]. This could be because women participating in this study were young; the average age was 46.34-years-old.

After cancer, the majority of women in this study (70.2%) confirmed that they continued to have sexual activity, with is consistent with the literature [14, 15, 16]. Although the frequency of sexual relations is lower after cancer, 60.7% reported that they have sexual relationships 1–2 times per month [5, 9, 12, 16].

The main sexuality problems are decreased sexual desire [6, 9,11, 14, 17, 18, 19], lubrication [5, 6, 11, 18, 19], excitation or arousal [10, 11, 16, 18, 19, 20], and penetration pain [12, 19].

Regarding the treatment and presence of sexual dysfunction, participants who had received chemotherapy presented more sexual problems that those who were not treated with chemotherapy [9, 21, 22]. However, these results are different from other studies reporting that chemotherapy is not associated with sexual dysfunction [6, 14]. Our data agree with Malinovszky et al. [23], in which premenopausal women with breast cancer who had received chemotherapy declared that they suffered decreased sexual pleasure and increased discomfort [23].

In our study, the majority of women were treated with hormonal therapy (75.7%) (e.g., aromatase inhibitors or tamoxifen), which led to early menopause. As found in other studies, the use of these drugs is related to reduced vaginal lubrication, decreased sexual satisfaction, and loss of sexual desire [6, 13, 17, 18, 24, 25].

The type of surgical intervention is associated with the presence of sexual dysfunction. Women with lumpectomy present fewer dysfunctions that those who have a unilateral mastectomy or a bilateral mastectomy [6, 9, 26, 27]. Women without breast reconstruction have the greatest probability of sexual dysfunction [28].

Women who were old at the age of diagnosis reported that they had more sexual problems [29, 30]. However, the young women after treatment had less sexual dysfunction, because their ovaries can continue to work properly. On the other hand, in premenopausal and perimenopausal women, ovarian function is usually affected for a long period following chemotherapy and hormonal therapy [31].

Civil status is associated with the presence of several sexual dysfunctions. Women without a partner do not have problems related to orgasm and penetration. Another finding is that women who have a steady partner but do not cohabitate have fewer problems related to desire, excitement, and lubrication compared to married women or couples living together. Although we found no study similar to ours, Morais et al. [14] reported that women without a steady partner have better sexual satisfaction due to multiple partners.

The study has some limitations. First, a cross-sectional design was used, so it is not possible to establish causal relationships. Second, a convenience sample was used. Finally, the online questionnaire prevents women from asking about any doubts they might have, and the analyzed data are self-reported information. However, on the other hand, the online questionnaire has the strength that the women can provide detailed answers about their sexuality more freely and without shame, which is not always possible in a face-to-face interview.

Furthermore, although the sample was recruited for convenience, it has a large size (more than 500 women). Also, we believe that the women can adequately respond to questions addressing their clinical situation. This is because, in Spain, the Health System is public and the physicians are obligated to give to their patients (in this case, women with breast cancer) a report that includes specific information about their type of cancer, type of surgery, and type of treatment.

Our findings highlight the impact of breast cancer treatments on female sexuality and the impact in women with breast cancer.

Based on our findings, it is necessary that health professionals recognize which variables influence sexual function in women with breast cancer. A multidisciplinary team should plan interventions to facilitate sexual adjustment.

Furthermore, women with breast cancer are capable of choosing whether to be sexually active or not. It is the duty of health professionals to guarantee that women with breast cancer and their partners receive accurate information about sexuality, treatment, and emotional support [32].

## Conclusion

Sexual function changes after breast cancer. Ninety percent of cases suffer from some type of sexual dysfunction. The most frequent problems are dysfunction due to penetration pain, lubrication, desire, and excitation. The type of surgical intervention, hormonal therapy, age, and civil status are associated with the presence of sexual dysfunction.

## Supporting information

S1 File Available data files. (SAV)

S2 File Questionnaire translated. (DOCX)

## References

[1] Ferlay J, Soerjomataram I, Dikshit R, Eser S, Mathers C, Rebelo M, et al Cancer incidence and mortality worldwide: sources, methods and major patterns in GLOBOCAN 2012. International journal of cancer. 2015;136(5):e359–86. 10.1002/ijc.29210 25220842

[2] Sociedad Española de Oncología Médica (SEOM). Las Cifras del Cáncer en España, 2017. Madrid: SEOM; 2017 Available from: http://www.seom.org/seomcms/images/stories/recursos/Las_cifras_del_cancer_en_Esp_2017.pdf

[3] Galceran J, Ameijide A, Carulla M, Mateos A, Quirós J, Rojas D, et al Cancer incidence in Spain, 2015. Clinical and Translational Oncology. 2017;19(7):799–825. 10.1007/s12094-016-1607-9 28093701

[4] Carmona-Torres JM, Cobo-Cuenca AI, Martín-Espinosa NM, Píriz-Campos RM, Laredo-Aguilera JA, Rodríguez-Borrego MA. Prevalence in the performance of mammographies in Spain: Analysis by Communities 2006–2014 and influencing factors. Atención Primaria. 2017 7 18 pii: S0212-6567(16)30539-X. 10.1016/j.aprim.2017.03.007 28732722 PMC6836949

[5] Kedde H, Van de Wiel H, Schultz WW, Wijsen C. Sexual dysfunction in young women with breast cancer. Supportive Care in Cancer. 2013;21(1):271–80. 10.1007/s00520-012-1521-9 22714701

[6] Panjari M, Bell RJ, Davis SR. Sexual function after breast cancer. The Journal of Sexual Medicine. 2011;8(1), 294–302. 10.1111/j.1743-6109.2010.02034.x 21199377

[7] Sadovsky R, Basson R, Krychman M, Morales AM, Schover L, Wang R, et al Cancer and sexual problems. The Journal of Sexual Medicine. 2010; 7(1 Pt 2):349–73. 10.1111/j.1743-6109.2009.01620.x 20092444

[8] Sánchez F, Conchillo MP, Valls JB, Llorens OG, Vicentee JA, de las Mulas ACM. Design and validation of the questionnaire on Women's Sexual Function (WSF). Atención Primaria. 2004; 34(6):286–92. 15491520 10.1016/S0212-6567(04)79497-4 PMC7669196

[9] Alicikus ZA, Gorken IB, Sen RC, Kentli S, Kinay M, Alanyali H, et al Psychosexual and body image aspects of quality of life in Turkish breast cancer patients: a comparison of breast conserving treatment and mastectomy. Tumori. 2009;95(2):212–218. 19579868 10.1177/030089160909500213

[10] Cavalheiro JAC, da Costa Bittelbrunn AC, Menke CH, Biazús JV, Xavier NL, Cericatto R, et al Sexual function and chemotherapy in postmenopausal women with breast cancer. BMC women's health. 2012;12(1):28 10.1186/1472-6874-12-28 22963155 PMC3460742

[11] Emilee G, Ussher J, Perz J. Sexuality after breast cancer: a review. Maturitas. 2010;66(4):397–407. 10.1016/j.maturitas.2010.03.027 20439140

[12] Ussher JM, Perz J, Gilbert E. Changes to sexual well-being and intimacy after breast cancer. Cancer nursing. 2012;35(6):456–65. 10.1097/NCC.0b013e3182395401 22222680

[13] Alder J, Zanetti R, Wight E, Urech C, Fink N, Bitzer J. Sexual dysfunction after premenopausal stage I and II breast cancer: do androgens play a role? The Journal of Sexual Medicine. 2008;5(8):1898–906. 10.1111/j.1743-6109.2008.00893.x 18554258

[14] Morais FD, Freitas‐Junior R, Rahal RMS, Gonzaga CMR. Sociodemographic and clinical factors affecting body image, sexual function and sexual satisfaction in women with breast cancer. Journal of clinical nursing. 2016;25(11–12):1557–65. 10.1111/jocn.13125 27139170

[15] Cairo Notari S, Favez N, Notari L, Panes‐Ruedin B, Antonini T, Delaloye JF. Women's experiences of sexual functioning in the early weeks of breast cancer treatment. European Journal of Cancer Care. 2018;27:e12607 10.1111/ecc.12607 29372622

[16] Ussher JM, Perz J, Gilbert E. Perceived causes and consequences of sexual changes after cancer for women and men: a mixed method study. BMC cancer. 2015;15:268 10.1186/s12885-015-1243-8 25885443 PMC4407322

[17] Biglia N, Moggio G, Peano E, Sgandurra P, Ponzone R, Nappi RE, et al Effects of surgical and adjuvant therapies for breast cancer on sexuality, cognitive functions, and body weight. The Journal of Sexual Medicine. 2010;7(5):1891–900. 10.1111/j.1743-6109.2010.01725.x 20233281

[18] Safarinejad MR, Shafiei N, Safarinejad S. Quality of life and sexual functioning in young women with early‐stage breast cancer 1 year after lumpectomy. Psycho‐Oncology. 2013;22(6):1242–8. 10.1002/pon.3130 22777952

[19] Wang F, Chen F, Huo X, Xu R, Wu L, Wang J, et al A neglected issue on sexual well-being following breast cancer diagnosis and treatment among Chinese women. PloS one. 2013;8(9):e74473 10.1371/journal.pone.0074473 24086349 PMC3783444

[20] Vaidakis D, Panoskaltsis T, Poulakaki N, Kouloura A, Kassanos D, Papadimitriou G, et al Female sexuality after female cancer treatment: a clinical issue. European journal of gynaecological oncology. 2014;35(6):635–40. 25556267

[21] Ganz PA, Kwan L, Stanton AL, Krupnick JL, Rowland JH, Meyerowitz BE, et al Quality of life at the end of primary treatment of breast cancer: first results from the moving beyond cancer randomized trial. Journal of the National Cancer Institute. 2004; 96(5):376–87. 14996859 10.1093/jnci/djh060

[22] Webber K, Mok K, Bennett B, Lloyd AR, Friedlander M, Juraskova I, et al If I am in the mood, I enjoy it: an exploration of cancer-related fatigue and sexual functioning in women with breast cancer. The oncologist. 2011;16(9):1333–44. 10.1634/theoncologist.2011-0100 21835897 PMC3228173

[23] Malinovszky K, Gould A, Foster E, Cameron D, Humphreys A, Crown J, et al Quality of life and sexual function after high-dose or conventional chemotherapy for high-risk breast cancer. British journal of cancer. 2006;95(12):1626–31. 10.1038/sj.bjc.6603454 17160080 PMC2360752

[24] Dizon DS, Suzin D, McIlvenna S. Sexual health as a survivorship issue for female cancer survivors. The oncologist. 2014;19(2):202–10. 10.1634/theoncologist.2013-0302 24396051 PMC3926787

[25] Ochsenkühn R, Hermelink K, Clayton AH, von Schönfeldt V, Gallwas J, Ditsch N, et al Menopausal status in breast cancer patients with past chemotherapy determines long‐term hypoactive sexual desire disorder. The Journal of Sexual Medicine. 2011;8(5):1486–94. 10.1111/j.1743-6109.2011.02220.x 21366876

[26] Aerts L, Christiaens M, Enzlin P, Neven P, Amant F. Sexual functioning in women after mastectomy versus breast conserving therapy for early-stage breast cancer: a prospective controlled study. The breast. 2014;23(5):629–36. 10.1016/j.breast.2014.06.012 25082211

[27] Raggio GA, Butryn ML, Arigo D, Mikorski R, Palmer SC. Prevalence and correlates of sexual morbidity in long-term breast cancer survivors. Psychology & health. 2014;29(6):632–50. 10.1080/08870446.2013.879136 24404999 PMC5521019

[28] Andrzejczak E, Markocka‐Mączka K, Lewandowski A. Partner relationships after mastectomy in women not offered breast reconstruction. Psycho‐Oncology. 2013;22(7):1653–7. 10.1002/pon.3197 23045167

[29] Lopes JdSO, Costa LL, Guimarães JV, Vieira F. The sexuality of women undergoing treatment for breast cancer. Enfermería global. 2016;15(3):350–406.

[30] Kirshbaum MN, Dent J, Stephenson J, Topping AE, Allinson V, McCoy M, et al Open access follow‐up care for early breast cancer: a randomised controlled quality of life analysis. European journal of cancer care. 2017;26(4):e12577 10.1111/ecc.12577 27717057 PMC5516199

[31] Burwell SR, Case LD, Kaelin C, Avis NE. Sexual problems in younger women after breast cancer surgery. Journal of Clinical Oncology. 2006;24(18):2815–2821. 10.1200/JCO.2005.04.2499 16782919

[32] Gilbert E, Perz J, Ussher JM. Talking about sex with health professionals: the experience of people with cancer and their partners. Eur J Cancer Care (Engl). 2016;25(2):280–93. 10.1111/ecc.12216 25040442

