# Supplementary material for: Sexual dysfunction in Spanish women with breast cancer
Source: PLoS One. 2018 Aug 31;13(8):e0203151. doi: 10.1371/journal.pone.0203151 (PMC6118366; doi:10.1371/journal.pone.0203151)
Supplement: S2 File — (DOCX) [file pone.0203151.s002.docx]

|  | Never | Rarely | Sometimes | Often | Almost always-  Always |
| --- | --- | --- | --- | --- | --- |
| 1. Have you had fantasies (thoughts, images ...) related to sexual activities during the last 4 weeks? |  |  |  |  |  |
| 2. Have you had any thoughts or desires to engage in any type of sexual activity during the last 4 weeks? |  |  |  |  |  |
| 3. Has it been easy for you to become sexually aroused during the last 4 weeks? |  |  |  |  |  |
| 4. During sexual activity in the last 4 weeks, when you have (or have) touched or caressed you have felt sexual excitement?, For example feeling of "getting going", desire to "advance more" in sexual activity |  |  |  |  |  |
| 5. During the past 4 weeks, when you felt sexually aroused, did you notice moisture and / or vaginal lubrication? |  |  |  |  |  |
| 6. During your sexual activity in the last four weeks, when you have been touched or caressed in the vagina and / or genital area, have you felt pain?  **Instructions**: If you have not felt pain, go to question 8 |  |  |  |  |  |
| 7a. During sexual activity in the last 4 weeks, vaginal penetration (of the penis, finger, object ...) could be done easily? |  |  |  |  |  |
| 7b. Indicate why there has been no vaginal penetration during the last 4 weeks |  |  |  |  |  |
| 8. At the idea or possibility of sexual activity during the last 4 weeks, have you felt fear, restlessness, anxiety ..........? |  |  |  |  |  |
| 9. Have you reached orgasm when you have maintained sexual activity, with or without penetration during the last 4 weeks? |  |  |  |  |  |
| 10. How many times have you been the one who has taken the initial steps to provoke a sexual encounter with another person in the last 4 weeks? |  |  |  |  |  |
| 11. Have you felt confident to communicate to your partner what you like or dislike in your sexual encounters during the last 4 weeks? |  |  |  |  |  |
| 12. How many times have you had sexual activity during the last 4 weeks? |  |  |  |  |  |
| 13. Did you enjoy when you performed sexual activity during the last 4 weeks? |  |  |  |  |  |
| 14. In general, in relation to your sex life during the last 4 weeks, have you felt satisfied? |  |  |  |  |  |

*Questionnaire on Women's Sexual Function (WSF)* of Sánchez et al. (2004)

Sánchez F, Conchillo MP, Valls JB, Llorens OG, Vicente JA, de las Mulas ACM. Design and validation of the questionnaire on Women's Sexual Function (WSF). Atención Primaria. 2004; 34(6):286-92. <http://www.elsevier.es/es-revista-atencion-primaria-27-articulo-diseno-validacion-del-cuestionario-funcion-13067028>

This questionnaire is use to evaluate the sexual function in the Spanish Health System Services <http://www.juntadeandalucia.es/servicioandaluzdesalud/library/plantillas/externa.asp?pag=/contenidos/gestioncalidad/CuestEnf/PT9_FuncSexMujer.pdf>
